# Autonomous demand and technical change: exploring the Kaldor–Verdoorn law on a global level

**DOI:** 10.1007/s40888-023-00294-y

**Published:** 2023-01-18

**Authors:** Matteo Deleidi, Claudia Fontanari, Santiago José Gahn

**Affiliations:** 1 Department of Political Sciences, University of Bari Aldo Moro, Bari, Italy; 2 Institute for Innovation and Public Purpose, University College London, London, UK; 3 Department of Economics, Roma Tre University, Rome, Italy

**Keywords:** Labour productivity, Autonomous demand, Panel SVAR, Penn World Table, Kaldor–Verdoorn, C33, E12, E24, O33, O47

## Abstract

This paper aims to explain labour productivity through the lens of a Kaldorian perspective. To assess the relationship between output, demand, capital accumulation, and labour productivity, we apply Panel Structural Vector Autoregressive (P-SVAR) modelling to a dataset of 52 countries observed over a long-time span as provided by the Penn World Table. Findings validate the Kaldorian perspective and show that demand shocks—measured by government expenditures and exports—produce positive and persistent effects on labour productivity. Findings are confirmed even when the full sample is broken down to consider developed and developing countries separately.

## Introduction

Usually regarded as one of the main causes of slacking economic growth, the prolonged stagnation of productivity in recent decades has been a concern for several advanced economies. This interpretation is grounded on a theoretical approach where economic growth is explained in terms of and limited by both the growth of the factors of production, labour and capital, and technical progress. From this perspective, there is a long-run path in which the system gravitates towards positions of full employment and the constraints to an increasing output are the growth in productive efficiency and the accumulation of productive resources. In a situation where all resources are fully utilised, labour productivity growth is interpreted as an improvement in technical efficiency and is ascribed exclusively to supply-side factors such as a change in endowments or production techniques.

By contrast, in a demand-led growth theoretical approach, no automatic mechanism ensures a tendency towards full employment of productive resources, and economic growth is interpreted as fundamentally determined by aggregate demand. In this approach, the actual path of the economy, which is determined by demand, influences the trajectory of its growth: supply conditions define the path of potential output in a way that is *not* independent of the actual path of output determined by demand. Consequently, labour productivity is seen as largely endogenous to aggregate demand, which thus constitutes a constraint on both output and productivity growth. As postulated by the Kaldor–Verdoorn law, a stable long-run relationship between output and labour productivity exists, whereby output growth determines productivity growth. Market expansion becomes a necessary condition for activating those technological and organisational factors which favour productivity growth, such as incentives to the introduction of organisational improvements and more efficient employment of inputs, changes in the sectoral composition of output and employment, static (linked to the indivisibility of the production process) as well as dynamic (involving innovative activities and learning-by-doing) economies of scale, and increased investment that incorporates technical progress (Kaldor, 1957, 1966; Verdoorn, 1949). Over the years, several studies have been carried out and have found empirical confirmation of a positive relationship moving from output to productivity (for a detailed review, see Deleidi et al., 2020b, 2021b; McCombie et al., 2002). The Kaldor–Verdoorn law thus constitutes empirical evidence for interpreting labour productivity dynamics as predominantly endogenous to aggregate demand. Specifically, in his analysis of the determinants of productivity growth from 1970 onwards, Kaldor (1975a) attributes a crucial role to the exogenous components of demand and, in particular, to export growth. The external demand for manufactured products is interpreted as an exogenous factor that can activate the cumulative expansion of manufacturing output and productivity (Kaldor, 1975a, 1977).^1^

Based on these premises, in this paper, we analyse the relationship described by the Kaldor–Verdoorn law by introducing the intuition developed by Kaldor (1975a) and thus focusing on the effects of autonomous demand components on labour productivity dynamics. To do this, we theoretically merge the standard Kaldor–Verdoorn law with a demand-led model of growth based on the notions of supermultiplier and autonomous components of demand (see Serrano, 1995; Freitas & Serrano, 2015). Empirically, we use advanced panel techniques based on Panel Structural Vector Autoregressive (P-SVAR) modelling (Pedroni, 2013) applied to a dataset provided by the Penn World Table (Feenstra et al., 2015) and composed by 3089 observations from 52 countries observed over a long-time span. This database provides us with relevant variables such as GDP, capital stock and average annual hours worked by persons engaged, which allow us to calculate the capital-labour ratio and labour productivity. The autonomous demand is calculated as the sum of government consumption expenditure and exports. The use of autonomous demand and its components along with P-SVAR techniques brings about a significant improvement in the recent empirical literature on the topic, making the identification of demand shocks and the evaluation of their effects on labour productivity possible (Deleidi et al., 2020b, 2021b). Models considering government expenditure and exports separately allow us to verify whether fiscal policy stimuli can affect innovation processes. To provide a complete picture of our findings, we split the dataset into two subgroups: ‘developed’ and ‘developing’ countries, following the United Nations classification (UN, 2020). This will allow us to evaluate how the effects on productivity produced by changes in demand can affect economies characterised by different stages of development differently. Our findings validate the Kaldor–Verdoorn law when considering the entire sample and breaking down the dataset into ‘developed’ and ‘developing’ countries. Additionally, we show that autonomous demand shocks positively affect labour productivity, and therefore expansionary fiscal policies can foster innovation.

The structure of the paper is as follows. In Sect. 2, we present the theoretical framework, while in Sect. 3, we review the empirical literature on the abovementioned determinants of labour productivity. In Sect. 4, we introduce data and methods used for empirical analysis. Section 5 is devoted to presenting our empirical findings, and Sect. 6 concludes.

## Theoretical background

The Kaldor–Verdoorn’s law is a well-known empirical relationship according to which there exists a stable long-run relationship between output and labour productivity, whereby output growth determines productivity growth (Deleidi & Mazzucato, 2019; Kaldor & Mirrlees, 1962; Kaldor, 1966, 1968; Lavoie, 2015; McCombie et al., 2002; Setterfield & Cornwall, 2002; Sylos Labini, 1984; Verdoorn, 1949). In this paradigm, technical progress is thus regarded as endogenously determined by output growth. According to Kaldor’s interpretation (Kaldor, 1957, 1966), the long-run relationship between output increase and productivity is due to: (1) the presence of static and dynamic economies of scale associated with division-of-labour and learning-by-doing processes, which in turn derive from an increased level of specialisation (Verdoorn, 1949); (2) increased investment embodying technical progress (Kaldor, 1957). Following a post-Keynesian perspective, both mechanisms originate from the growth of aggregate demand, which thereby positively affects labour productivity dynamics.

The interpretation of Kaldor–Verdoorn’s law also has essential theoretical reference in the analysis of Adam Smith, describing the phenomenon of increasing returns as the result of an increase in the division of labour. Smith (1776) argued that the growth of labour productivity is due to an increase in the division of labour and productive specialisation. Both these mechanisms originate from the expansion of the market. Processes of productive specialisation can take place both *between* an increasing number of firms, favouring productive diversification and the creation of new firms or industries, and *within* firms through an increased fragmentation of operations, favouring processes of concentration and growth in size (see Kaldor, 1966; Smith, 1776; Sylos Labini, 1984). Hence, static and dynamic economies of scale are associated with increasing returns to scale, whereby increased production leads to savings in input per unit of output (Kaldor, 1966). According to Kaldor, the development of economies of scale is peculiar to the manufacturing sector, which he considers to be the engine of economic growth. Dynamic economies of scale enhance returns as output increases through the accumulation of experience and knowledge, fostering innovative activity (Arrow, 1971). Static economies of scale, on the other hand, operate even in the absence of technological progress: the return per unit of labour grows as the size of production increases for technical and organisational reasons due, for example, to the presence of indivisibility in the production process (see Kaldor, 1957, 1966; Kaldor & Mirrlees, 1962).

It is worth noticing that interpreting productivity growth as endogenously determined by output growth does not exclude that a reverse influence might also exist, namely from productivity growth to output growth. Indeed, Kaldor’s analysis highlights the existence of a virtuous circle linked to a cumulative causation process, according to which output growth favours productivity growth, which in turn leads to further increases in output through the positive effect of productivity on external competitiveness (see Kaldor, 1970, 1972). Increased competitiveness would favour exports, which, according to Kaldor, are the main driver of economic growth as a truly autonomous component of demand (Palumbo, 2009). Moreover, the rise in productivity can reduce the propensity to import and, by lowering relative prices, increase the propensity to consume (Cesaratto et al., 2003). Exports would thus have the dual role of fuelling the virtuous circle of growth and, by financing imports, relieving the possible constraint that trade imbalances can place on output growth (Thirlwall, 1979).

The relationship outlined by the mechanisms mentioned above is shown, in its simplest version, in Eq. (1), generally known as Verdoorn’s law:

1 $$\dot{lp}=\alpha +\eta \dot{y}$$

where $\dot{lp}$ represents the rate of growth of labour productivity; $\dot{y}$ is the rate of growth of output; $\alpha$ represents the pace of exogenous technical progress; and $\eta$ measures the relationship between $\dot{y}$ and $\dot{lp}$. From now on, we shall refer to the $\eta$ parameter as the *Verdoorn effect*.

When addressing the second mechanism concerning the effect of technical progress embodied in newly installed capital goods,^2^ Kaldor (1957) includes the capital-labour ratio in his analysis. As shown in Eq. (2), Kaldor’s original technical progress function postulates the existence of a positive relationship between the growth rate of labour productivity ($\dot{lp}$) and the growth rate of the capital-labour ratio ($\dot{k}$):

2 $$\dot{lp}=\delta +\lambda \dot{k}$$

Since “the use of more capital per worker inevitably entails the introduction of superior techniques” (Kaldor, 1957, p. 595), the growth of the capital-labour ratio aims to capture the effect on technical progress of “the speed with which innovations are introduced” (Lavoie, 2015, p. 429). We shall thus refer to the $\lambda$ parameter as the *capital accumulation effect*.

Starting with Michl (1985), the two effects on labour productivity growth described are combined in a single relationship reported in Eq. (3) (see also Antenucci et al., 2020; Deleidi et al., 2021b):

3 $$\dot{lp}=\epsilon +\eta \dot{y}+\lambda \dot{k}$$

where labour productivity growth ($\dot{lp}$) is affected by both the rate of growth of output ($\dot{y}$) and the rate of growth of the capital − labour ratio ($\dot{k}$). Equation (3) thus combines the Verdoorn effect ($\eta$) with the effect of capital accumulation ($\lambda$) described in Kaldor’s technical progress function (Kaldor, 1957).

The Kaldor–Verdoorn law has been extensively studied within the post-Keynesian or demand-led growth theoretical framework, which underlines the central role of demand in determining both output and productivity growth. Therefore, in this perspective, the Verdoorn effect formalised in the Kaldor–Verdoorn law can be interpreted as the effect of changes in aggregate demand on productivity. Recent theoretical contributions have nested the Kaldor–Verdoorn law inside the supermultiplier model of growth (Deleidi & Mazzucato, 2019; Nah & Lavoie, 2019). According to the supermultiplier model, GDP growth is driven both in the short- and long-run by the evolution of the non-capacity creating autonomous components of demand, namely export, government expenditure, and autonomous consumption (Cesaratto et al., 2003).^3^ These components are defined as autonomous and non-capacity creating since they neither depend on the current level of income nor directly affect productive capacity (Serrano, 1995). Specifically, government expenditure is determined by fiscal policy decisions; exports depend on the level of foreign demand; and autonomous consumption is financed through bank loans created through an endogenous money creation process or accumulated wealth (Barbieri Góes & Deleidi, 2022).^4^ Following the supermultiplier model of growth, the relationship between the rate of growth of output ($\dot{y}$) and the autonomous components of demand ($\dot{z}$) can be written as in Eq. (4):

4 $$\dot{y}=m\dot{z}$$

where $m$ is the supermultiplier, $\dot{y}$ is the rate of growth of output, and $\dot{z}$ is the rate of growth of autonomous components of demand. By substituting Eq. (4) into Eq. (3), we obtain Eq. (5)

5 $$\dot{lp}=\epsilon +\eta (m\dot{z})+\lambda \dot{k}$$

where labour productivity growth ($\dot{lp}$) is determined by both the rate of growth of autonomous components of demand ($\dot{z}$) and the rate of growth of the capital–labour ratio ($\dot{k}$). It is worth noting that the combination of the supermultiplier model with the Kaldor–Verdoorn law is entirely in line with the Kaldorian perspective: “I would now place more, rather than less, emphasis on the exogenous components of demand, and in particular on the role of exports, in determining the trend rate of productivity growth in the United Kingdom in relation to other industrially advanced countries” (Kaldor 1975a, p. 896).^5^ Furthermore, Eq. (5) also considers the level effects that different supermultipliers may produce on the average growth rate of output and labour productivity, by affecting permanently their levels. In other words, while the size of the supermultipliers does not affect the trend growth rate of output and productivity, different supermultipliers can produce different level effects during the adjustment process from a fully adjusted position to another one, thus producing different *average* growth rates of output and productivity. Since the size of the market is determined both by the rate of growth of autonomous components of demand ($\dot{z}$) and the magnitude of the supermultiplier ($m$), then, assuming a constant $\dot{z}$, the greater $m$ the larger the expansion of the market.

In the current paper, by applying P-SVAR techniques to a panel dataset of 52 countries observed over a long-time span, we validate Eqs. (3) and (5). When considering Eq. (5), we also estimate the effect of different components of autonomous demand by breaking down autonomous expenditures into government spending and exports. Finally, as explained in the following sections, the use of autonomous demand will allow us to overcome potential empirical issues related to the identification of demand shocks.

## Empirical literature

Although the empirical literature on the factors enhancing productivity growth is extremely vast, we focus on empirical evidence in favour of a positive effect on labour productivity growth associated with output growth and capital accumulation. After the initial estimations by Verdoorn (1949), who estimated a Verdoorn effect of 0.45, and by Kaldor (1966), who assessed a dynamic version of Verdoorn’s law for which an additional percentage point of output growth leads to a 0.5% increase in productivity, several studies contributed to validate the Verdoorn law. An extensive review of those empirical investigations can be found in McCombie (1983), Thirlwall (1983), and McCombie et al. (2002).

In recent contributions, the Kaldor–Verdoorn law has been empirically verified both in country-specific studies and through cross-country analyses. Among the formers, Bianchi (2002), Ofria (2009), and Forges Davanzati et al. (2019) estimated the Verdoorn coefficient for the Italian economy at between 0.5 and 0.7; Apergis and Zikos (2003) verified the Verdoorn law for Greece; Castiglione (2011) estimated the Verdoorn coefficient for the US manufacturing sector through a cointegration analysis; Millemaci and Ofria (2014) validated the long-run dynamic Verdoorn law for the manufacturing industry in several developed economies. Clavijo-Cortes (2021) found a Verdoorn coefficient varying between 0.1 and 0.37 for the US economy. Recently, Iasco Pereira et al. (2021) have found that results are positive and significant even when controlling for institutions and inequality in the case of Brazil (at the municipal level). They tested the direct and indirect impacts of institutions on productivity growth, controlling for human and physical capital, as well as for demand growth. They found that in municipalities with inclusive institutions and higher human capital, productivity growth responds more strongly to demand growth. Nassif et al. (2022) find results that validate the Kaldor–Verdoorn law also for the case of Brazil. Concerning panel analysis, Knell (2004) estimated a Verdoorn coefficient of 0.53 with respect to the manufacturing sectors of 12 industrial countries during the 1990s; Tridico and Pariboni (2018) estimated a Verdoorn effect of 0.36 in a panel of OECD countries. Similarly, Magacho and McCombie (2017) found Verdoorn coefficient around 0.5 in a panel of manufacturing industries; Dosi and Yu (2019), by making use of the Generalized Method of Moments (GMM) estimator on a Chinese firm-level database observed for the 1998–2007 period, estimated a significant long-run Kaldor–Verdoorn effect of 0.599. Gabrisch (2021) applied the model to a panel of Central and Eastern European countries; the author finds that slower productivity growth is not due to ‘adverse technological progress’ but to weakening external and domestic demand. Deleidi et al., (2020a, 2020b) validated the Verdoorn law for the manufacturing sector in nine Eurozone countries by using an ARDL model and autonomous components of demand, while Carnevali et al. (2020) estimated a positive ‘Smith effect’ in the Euro area manufacturing industries within the context of Sylos Labini’s productivity equation (Sylos Labini, 1984).^6^

Regarding the effect of capital per unit of labour on productivity dynamics, several works have demonstrated the positive role of capital accumulation in fostering productivity. In this paradigm, labour productivity growth is typically decomposed into the contributions of two components: capital deepening and total factor productivity. Among these, the process of increasing capital per unit of labour has a positive and significant effect on productivity according to a number of authors, including Kumar and Russell (2002), who estimate a contribution of capital accumulation to productivity growth of 77% for a panel of 57 countries, as well as Jorgenson et al. (2008) and Foda (2017), who estimate a contribution of capital accumulation to productivity growth for the US economy of about 53 and 45%, respectively. Sichel and Oliner (2002) analyse the influence of ICT investment on productivity growth in the post-1995 period in the US nonfarm business sector and they find a contribution of ICT to the growth of labour productivity of 42% and a contribution of other forms of capital of 7%. Van Ark et al. (2008) evaluate the productivity gap between Europe and the US estimating a positive contribution of capital service per hour worked to average productivity growth of 1 and 1.3% points for the EU and the US. Borsato and Lorentz (2022) incorporate the notion of robotisation into the analysis; the authors find that a higher degree of robotisation strengthens the mechanism that links labour productivity growth at the industrial level to the macroeconomic level.

Closely related to the purposes of this paper are the results obtained by Michl (1985), who was the first to estimate an extended version of Kaldor’s technical progress function, finding a Verdoorn coefficient of 0.54 and a capital accumulation coefficient of 0.40 for eight advanced countries for the period 1950–1983. More recently, Antenucci et al. (2020) have estimated a positive capital accumulation coefficient and a positive Verdoorn coefficient for G7 countries, while Deleidi et al. (2021b) have obtained confirmation of the extended version of Kaldor’s technical progress function at the Italian regional level.^7^

To sum up, the empirical literature largely confirms the existence of a positive effect of output growth and capital accumulation on labour productivity growth. Estimated coefficients vary depending on the methods used and the temporal-spatial level of the analysis. Following part of the recent literature (Antenucci et al., 2020; Deleidi et al., 2020b, 2021b), in this paper, we use P-SVAR modelling to validate Eqs. (3) and (5). The employed method allows us to consider and overcome a long-debated problem in the relevant literature, namely the endogeneity of investment to output growth. The novelty of this paper lies in the use of autonomous components of aggregate demand as a proxy for the Verdoorn effect, and the application of P-SVAR techniques to a dataset of 52 countries. The use of demand components—namely government expenditure, export, and their sum—will allow us to identify demand shocks using P-SVAR techniques, that is shocks that can be associated with “a distinct economic interpretation” (Kilian & Lütkepohl, 2017, p. 209), and then assess whether these shocks produce any effects on labour productivity. The identification of demand shocks using autonomous components of demand and P-SVAR modelling is particularly relevant in this field of research since it would allow us to solve a twofold shortcoming in the considered empirical literature. First, the use of structural models to validate the Kaldor–Verdoorn law (Antenucci et al., 2020; Deleidi et al., 2021b), employing output growth rather than autonomous demand components, does not provide a meaningful economic interpretation of identified shocks because shocks associated with output can be theoretically ascribed to both supply and demand factors. Secondly, introducing demand components in a single equation modelling (Deleidi et al., 2020a, 2020b) implies assuming the exogeneity of autonomous demand components, which would allow us to neither estimate structural shocks nor assess the dynamic evolution of demand shocks on labour productivity dynamics and therefore assess its persistence. The current paper aims to improve the recent empirical analysis by overcoming the limitations mentioned above.

## Data and methods

### Data

The source of all our data is the Penn World Table v. 10.0 (Feenstra et al., 2015). From the whole database, we set a minimum limit of 30 observations per country, thus reducing the usable data to a set of 52 countries.^8^ From this database, we can construct the GDP level ($Y$), government consumption ($G$) and exports ($EXP$). In line with the supermultiplier literature, we sum $G$ and $EXP$ to obtain autonomous expenditure ($Z$). On the other hand, we construct a series of the capital-labour ratio ($k$) and labour productivity ($LP$). Both the capital-labour ratio and productivity are built considering the hours worked (and not the number of workers). In this way, we can adjust the series to exclude short-run cyclical fluctuations due to changes in capacity utilisation from $LP$ and $k$, typically described by the mechanism underlying Okun’s law. According to Okun’s law, an increase in output leads to productivity gains in cyclical phases of the economy because the change in the degree of capacity utilisation gives firms an incentive to vary the working time of existing workers rather than the number of workers.^9^ All variables are transformed into logarithms. Additional details are reported in Appendix A.

To provide a complete picture, we split the dataset into developed ($n=25$) and developing ($n=27$) countries using the United Nations methodology (UN, 2020, pp. 165–166). Of the 3089 annual observations, 1625 refer to developed countries, whereas 1464 observations refer to developing ones. Using this dataset—which considers a set of heterogeneous countries—we can determine how the various sources of demand differently affect productivity growth in economies at different stages of development.

### Methods

To assess the validity of the Kaldor–Verdoorn law, we make use of the Panel Structural Vector Autoregressive (P-SVAR) methodology (Pedroni, 2013). This usefully considers responses to both idiosyncratic and common shocks while permitting full cross member heterogeneity of the response dynamics (Pedroni, 2013, p. 180). The advantage of this methodology is that it allows us to consider the existing heterogeneity within individual countries of the panel and cross-sectional dependence that is likely to arise from the fact that individual countries of the sample respond not only to their own member-specific idiosyncratic shocks but also to shocks that are shared across countries within the panel (Pedroni, 2013, p. 181).^10^

A P-SVAR can be summarised as in Eq. (6):

6 $${{B}_{0i}x}_{i,t}={B}_{i}\left(L\right){x}_{i,t-n}+{w}_{i,t}$$

where ${B}_{0i}$ is the matrix of contemporaneous coefficients, $x$ is the vector of considered variables, ${B}_{i}(L)$ is the matrix of lagged coefficients, and ${w}_{i,t}$ is the vector of estimated structural shocks.^11^ P-SVAR modelling allows us to estimate ${w}_{i,t}$ by imposing suitable restrictions on the ${B}_{0i}$ matrix derived from the considered economic theory (Kilian & Lütkepohl, 2017). Once restrictions are imposed and structural shocks estimated, impulse response functions (IRFs) are computed to evaluate the dynamic effects produced by a shock on the variables included in the model. IRFs are reported with 95% confidence interval bands estimated by bootstrapping standard errors. All models include two lags for each variable estimated through the GTOS (general-to-specific) criteria (Pedroni, 2013). Additionally, we estimate the cumulative effects computed by dividing the cumulated response of labour productivity to the corresponding impulses (Deleidi et al., 2021a; Spilimbergo et al., 2009). Since models are in log-level, estimated IRFs and cumulative effects are elasticities.

We estimate three different models. In Model 1, vector $$x$$ includes $$[Y; k; LP]$$, namely variables reported in Eq. (3), Sect. 2. However, as argued in Sect. 3, the use of GDP as a determinant of productivity cannot shed light on an important point backed by several supporters of the Kaldor–Verdoorn law: whether demand shocks affect labour productivity dynamics. Indeed, the shock extrapolated by the output equation cannot be directly associated with a demand shock because supply factors may also determine it. To overcome this issue, we use variables that can be theoretically associated with a demand shock. To do this, the vast literature on fiscal policy (see, among others, Auerbach & Gorodnichenko, 2012; Blanchard & Perotti, 2002; Deleidi et al., 2020a, 2021a, 2021c), as well as the one on the supermultiplier (Barbieri Góes & Deleidi, 2022; Cesaratto et al., 2003; Freitas & Serrano, 2015; Serrano, 1995) allow us to define a demand shock. Specifically, in our models, demand shocks are identified employing the notion of autonomous demand ($$Z$$), government expenditure ($$G)$$, and export ($$EXP$$). Once innovations to demand are obtained, we aim to assess the effect of $$Z$$ and its components ($$G$$ and $$EXP$$) on productivity dynamics. To do this, we estimate Models 2 and 3. While Model 2 includes $$[Z; k; LP]$$ and output $$Y$$ is replaced by autonomous demand $$Z$$, Model 3 considers $$G$$ and $$EXP$$ separately. Indeed, Model 3 includes the following variables: $$[EXP;G; k; LP]$$.

The theoretical intuition developed by Michl (1985) and the identification strategies employed by Antenucci et al. (2020) and Deleidi et al. (2021b) are used to estimate Models 1, 2, and 3. Specifically, structural shocks are obtained using short-run restrictions and a Cholesky factorisation. In the case of Model 1, we assume the identification summarised in (7) where ‘$-$’ indicates an unrestricted parameter and ‘0’ represents a zero restriction:

7 $$\mathbf{Model\,1{:}}\quad {{B}_{0i}y}_{i,t}=\left[\begin{array}{ccc}-& 0& 0\\ -& -& 0\\ -& -& -\end{array}\right]\left[\begin{array}{l}{Y}_{i,t}\\ {k}_{i,t}\\ {LP}_{i,t}\end{array}\right]$$

The identification reported in Eq. (7) assumes that GDP affects labour productivity and the capital-labour ratio within the contemporaneous relationship. On the contrary, the output is not affected by labour productivity or the capital-labour ratio within the contemporaneous relationship. In the last row of Eq. (7), labour productivity is assumed to be affected by both the capital-labour ratio and output dynamics within the contemporaneous relationship (see also Eq. (3) in Sect. 2).^12^

When moving to the identification of Model 2, output ($Y$) is replaced by autonomous demand ($Z$). In line with the supermultiplier literature considering autonomous demand as the driver of GDP and labour productivity growth (Deleidi & Mazzucato, 2019), $Z$ is assumed independent from labour productivity and the capital-labour ratio within the contemporaneous observation, while $k$ and $LP$ are ordered as in Eq. 7.

8 $$\mathbf{Model\;2{:}}\quad {{B}_{0i}y}_{i,t}=\left[\begin{array}{ccc}-& 0& 0\\ -& -& 0\\ -& -& -\end{array}\right]\left[\begin{array}{c}{Z}_{i,t}\\ {k}_{i,t}\\ {LP}_{i,t}\end{array}\right]$$

Finally, as shown in Eq. (9), the identification employed in Model 3 is similar to the one used in Model 2. Following Barbieri Góes and Deleidi (2022), we assume that export ($EXP$) is more exogenous than government expenditure ($G$) within the contemporaneous relationship. However, to provide a clear picture, a robustness check is carried out by assuming an additional identification for Model 3 where government expenditure ($G$) is considered more exogenous than export ($EXP$) within the contemporaneous relationship. Findings and the corresponding identification strategy used for this robustness check are reported in Appendix B.

9 $$\mathbf{Model\;3{:}} \quad {{B}_{0i}y}_{i,t}=\left[\begin{array}{cccc}-& 0& 0& 0\\ -& -& 0& 0\\ -& -& -& 0\\ -& -& -& -\end{array}\right]\left[\begin{array}{c}{EXP}_{i,t}\\ {G}_{i,t}\\ {k}_{i,t}\\ {LP}_{i,t}\end{array}\right]$$

## Findings

In this section, we plot the IRFs estimated for Models 1, 2 and 3. In particular, we report findings of estimated models by showing both IRFs and cumulative effects.

**Fig. 1:**
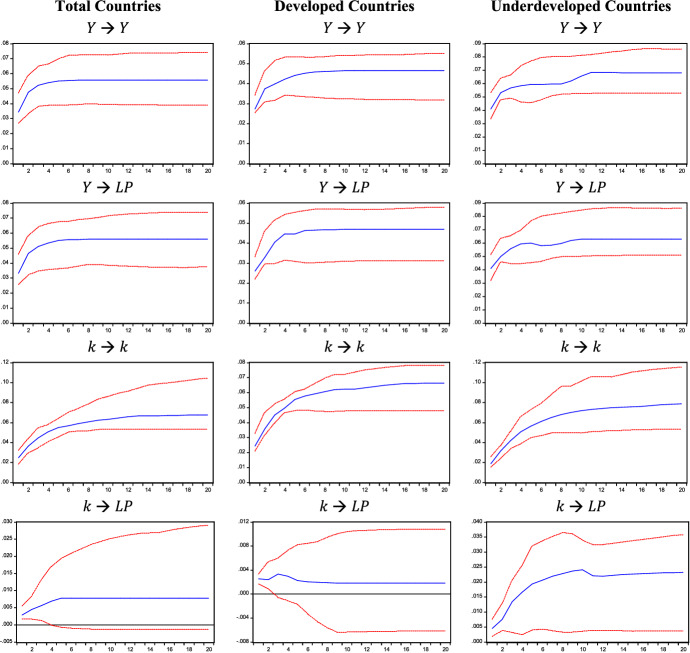
Impulse response functions (IRFs), Model 1. IRFs are reported with two standard error bounds (95% confidence interval)

**Fig. 2:**
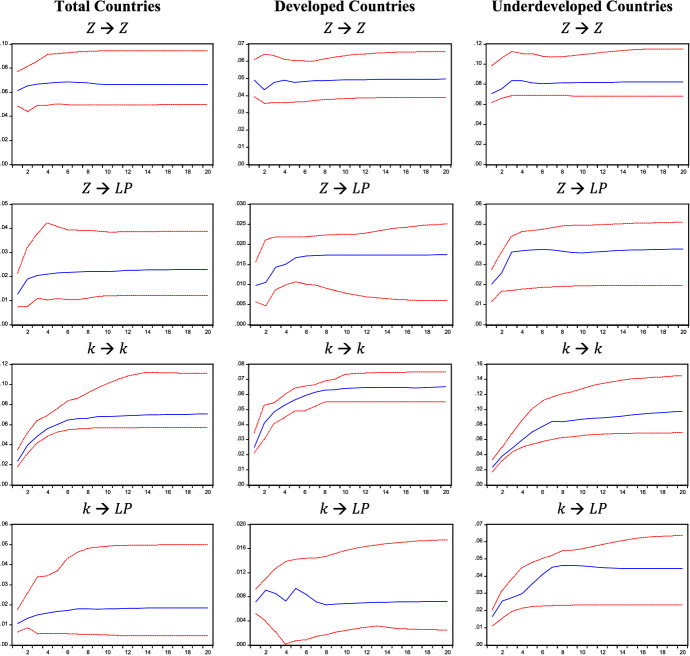
Impulse response functions (IRFs), Model 2. IRFs are reported with two standard error bounds (95% confidence interval)

**Fig. 3:**
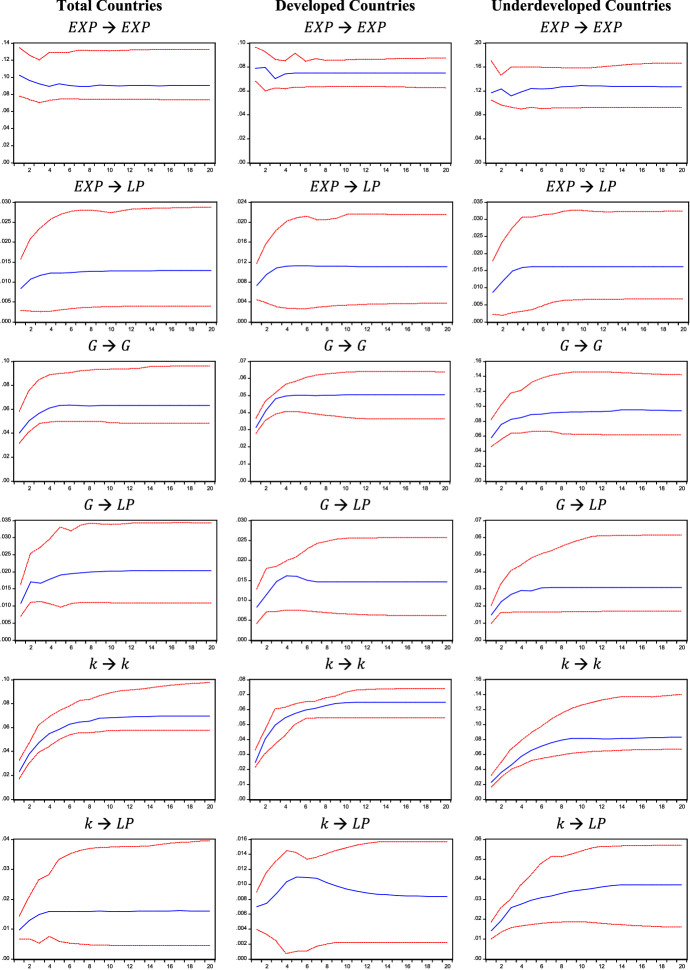
Impulse response functions (IRFs), Model 3. IRFs are reported with two standard error bounds (95% confidence interval)

IRFs are reported in Figs. 1, 2, and 3 and correspond to the estimates obtained for Model 1, 2, and 3, respectively. IRFs are highly persistent as they remain positive throughout the whole twenty-year period. When considering the Verdoorn effect, IRFs show that a rise in output ($Y$ in Fig. 1), autonomous demand ($Z$ in Fig. 2), and its components considered separately ($G$ and $EXP$ in Fig. 3) produces long-lasting effects on labour productivity ($LP$).

The persistence of these effects is also detected when analysing the capital accumulation effect. Indeed, an increase in capital-labour ratio $k$ leads to a persistent rise in labour productivity in Model 1, 2 and 3 (see Figs. 1, 2, and 3). Such evidence is also confirmed when breaking down the sample into developed and developing countries.

The cumulative effects are reported in Table 1. When considering all countries in Model 1, the Verdoorn effect is almost equal to 1%, whereas the capital accumulation effect is lower and equal to 0.138% on average. This means that a 1% change in output leads to a roughly proportional increase in labour productivity, while 1% increase in the capital-labour ratio raises labour productivity by 0.138%. By replacing the growth of output with the growth of autonomous demand ($Z$) in Model 2, the Verdoorn effect falls compared to Model 1, as a 1% increase in demand ($Z$) is followed by a labour productivity increase of 0.323% on average. Conversely, the capital accumulation effect estimated for Model 2 is higher than that obtained for Model 1: a unitary rise in the capital-labour ratio raises labour productivity by 0.281% on average. One possible explanation for the lower effect of autonomous demand could lie in the fact that ($Z$) excludes the impact of investment, which is instead partially captured by the growth of output; this would also explain the lower coefficient of the capital-labour ratio in Model 1, where the Verdoorn coefficient would partially capture the effect of embodied technical progress. The size of the estimated coefficient for the capital accumulation effect remains nearly unchanged in Model 3, where autonomous demand ($Z$) is broken down into government expenditure and export. When evaluating the effect of different demand components, government expenditure ($G$) produces larger effects on labour productivity than export ($EXP$) in all samples, assuming an average value of 0.314 and 0.135%, respectively. These results shed light on the role of autonomous demand components in determining productivity growth: while Kaldor (1977) emphasised the role of exports as the main driver, our results show a considerably higher impact of government spending. Findings obtained for Model 3 are also robust to a different identification strategy. Indeed, the IRFs and cumulative effects reported in Appendix B are in line with the results reported in this section.

**Table 1:** Cumulative effects

	1 year	5 years	10 years	20 years	Average
**Model 1**					
All countries					
$Y$	**0.970**	**1.059**	**1.030**	**1.026**	1.022
$k$	**0.124**	0.142	0.137	0.145	0.138
Developed					
$Y$	**0.951**	**1.009**	**1.009**	**1.010**	1.004
$k$	**0.102**	0.040	0.029	0.027	0.039
Developing					
$Y$	**1.002**	**1.009**	**0.957**	**0.922**	0.953
$k$	**0.243**	**0.343**	**0.336**	**0.293**	0.306
**Model 2**					
All countries					
$Z$	**0.206**	**0.314**	**0.333**	**0.345**	0.323
$k$	**0.456**	**0.275**	**0.264**	**0.262**	0.281
Developed					
$Z$	**0.199**	**0.349**	**0.353**	**0.350**	0.334
$k$	**0.288**	**0.166**	**0.107**	**0.111**	0.133
Developing					
$Z$	**0.284**	**0.456**	**0.436**	**0.457**	0.436
$k$	**0.712**	**0.504**	**0.527**	**0.457**	0.521
**Model 3**					
All countries					
$EXP$	**0.081**	**0.132**	**0.141**	**0.142**	0.135
$G$	**0.268**	**0.301**	**0.319**	**0.321**	0.314
$k$	**0.423**	**0.270**	**0.232**	**0.230**	0.258
Developed					
$EXP$	**0.092**	**0.150**	**0.148**	**0.148**	0.144
$G$	**0.261**	**0.319**	**0.289**	**0.290**	0.292
$k$	**0.284**	**0.190**	**0.145**	**0.128**	0.158
Developing					
$EXP$	**0.074**	**0.130**	**0.125**	**0.127**	0.123
$G$	**0.253**	**0.326**	**0.334**	**0.329**	0.325
$k$	**0.627**	**0.452**	**0.424**	**0.446**	0.463

Models 1, 2 and 3. Significant effects are in bold

When breaking down the sample into developed and developing countries, the estimates of Model 1 confirm a Verdoorn effect of around 1% for both subgroups. However, as autonomous demand is introduced in Model 2, results show that demand shocks produce different effects on the two groups of countries: while in developed countries, an increase in demand produces an average response of labour productivity of 0.334%, in developing countries such a response is equal to 0.436%. This could result from different stages of development; possibly, during the early stages of development, where sectors such as textiles are developing, productivity gains may be higher in these sectors (Magacho & McCombie, 2018). Ultimately, this result could be a consequence of the composition of output, something that should be analysed at the sectoral level and is beyond the scope of this paper. Going further, Model 3 allows us to observe the differences between countries according to the source of demand. As we have seen, on average, the impact of government spending is more important than exports for labour productivity growth, and this is true for both developed and developing countries. The impact of public spending on output and productivity growth is in line with our theoretical assumptions, whereby the government, by stimulating demand, creates room for market size to increase and this allows economies of scale to deepen, leading to productivity growth. Comparing the two groups, we observe that the average effect of government expenditure on productivity is greater in developing countries, while the average impact of export is greater in developed ones. The latter result is in line with Kaldor’s idea that exports are especially crucial to those countries that have a more advanced manufacturing sector and which, because of this, would be able to offer industrial products at lower costs, attract more demand for their manufactured goods and, thus, gain further productivity benefits (Kaldor, 1975b; Palumbo, 2009).^13^ Finally, the capital accumulation effect is larger in developing countries than in developed ones in all estimated models. Focusing on Model 2 and 3, the impact of capital accumulation ranges between 0.463 and 0.521% for developing countries and between 0.133 and 0.168% for developed countries. The larger effect of capital accumulation in developing countries may be related to a composition effect of investment. Indeed, this larger coefficient could capture the impulse, in developing countries, to adopt production methods with a higher capital endowment per worker, which stimulates higher productivity growth (as in the case of the manufacturing sector), while in developed countries a larger share of investment is directed to services where productivity gains are smaller. Furthermore, it is worth noticing that, in Model 2, demand shocks produce greater effects than capital accumulation on productivity when considering developed countries, while developing countries experience a slightly higher relevance of the capital accumulation effect. Model 3 shows that the most relevant impact on productivity for developed countries is the one generated by government expenditure, while it is again the accumulation of capital for developing countries.

## Conclusions and policy implications

The slowdown in productivity growth during the last decades is one of the most important issues to be explained and solved by economists and policymakers nowadays. In neoclassical growth models, productivity growth is considered one of the main drivers of long-run economic growth. According to this strand of thought, productivity and then economic growth are stimulated by the so-called supply-side factors, such as endowments and techniques of production. On the contrary, in demand-led growth models and notably the Kaldor–Verdoorn law (Kaldor, 1966; Verdoorn, 1949), the relationship between labour productivity and economic growth is reversed: a more sustained output growth—driven by aggregate demand expansion—leads to an increase in labour productivity. In this framework, the expansion of the market, determined by higher demand and thus by a higher level of production, stimulates innovation through: (1) incentives to improve organisational aspects and use inputs more efficiently; (2) changes in the sectoral composition of output and employment; (3) static and dynamic economies of scale; and (4) growth in investment embodying more advanced technological goods.

In the current paper, we have endorsed a Kaldorian perspective from a theoretical and empirical standpoint. Theoretically, we have followed the intuition developed by Kaldor (1975a), according to whom the exogenous components of demand play a crucial role in determining productivity trends. Accordingly, we have used the supermultiplier model of growth to formalise how autonomous demand and its components can exert positive and long-lasting effects on labour productivity. Empirically, we have employed advanced panel techniques based on a Panel Structural Vector Autoregressive (P-SVAR) model (Pedroni, 2013) to calculate Impulse Response Functions (IRFs) and quantify the dynamic effects of capital-labour ratio, output, autonomous demand and its components on labour productivity. To do this, we have applied P-SVAR modelling to a dataset provided by the Penn World Table composed of 3089 observations, divided into 52 countries observed over a long-time span. We have estimated three different models. In Model 1, we have assessed the standard Kaldor–Verdoorn law augmented by the capital-labour ratio and—with the introduction of autonomous demand in Model 2 and its breakdown into government expenditure and exports in Model 3—we have also been able to verify whether demand and fiscal policy shocks can exert positive and persistent effects on labour productivity dynamics. Additionally, our models have also been estimated on sub-samples considering ‘developed’ and ‘developing’ countries separately to provide a complete picture.

Findings have validated the Kaldor–Verdoorn law for the full sample and when the 52 considered nations are classified as ‘developed’ and ‘developing’ countries. We have shown that an autonomous demand shock positively affects labour productivity. Furthermore, when autonomous demand is broken down into government expenditure and export, government expenditure ($G$) produces larger effects on labour productivity than export ($EXP$), assuming an average value of 0.314 and 0.135%, respectively, both when the full sample is considered and when developed and developing countries are considered separately. It is worth noticing that, when comparing the two groups of countries, the effect produced by export is relatively higher in developed countries than in developing ones. In contrast, government expenditure exerts greater effects on productivity in developing countries than in developed ones. Finally, the capital accumulation effect positively influences labour productivity in all the estimated models. This effect is even higher than the one produced by demand shocks in developing countries. When looking at developed countries, the impact of demand on productivity—especially the one determined by government expenditure—is always predominant compared to the capital accumulation effect.

In conclusion, demand management policy seems to represent an appropriate tool to stimulate innovation and labour productivity. One of the main policy implications of our findings is that public expenditure and particularly expansionary fiscal policies can boost innovation and labour productivity. Thus, our results are in line with some of the policy prescriptions advocated in the recent *Fiscal Monitor* released by the International Monetary Fund (IMF, 2020): a well-designed fiscal policy plan can stimulate the structural transformation of the economy and foster productivity growth. This result is particularly relevant nowadays if policy prescriptions aimed at allowing countries to emerge from the stagnation generated by the COVID-19 pandemic and the one experienced after the Great Recession are to be formulated.

## Figures and Tables

**Fig. 4:**
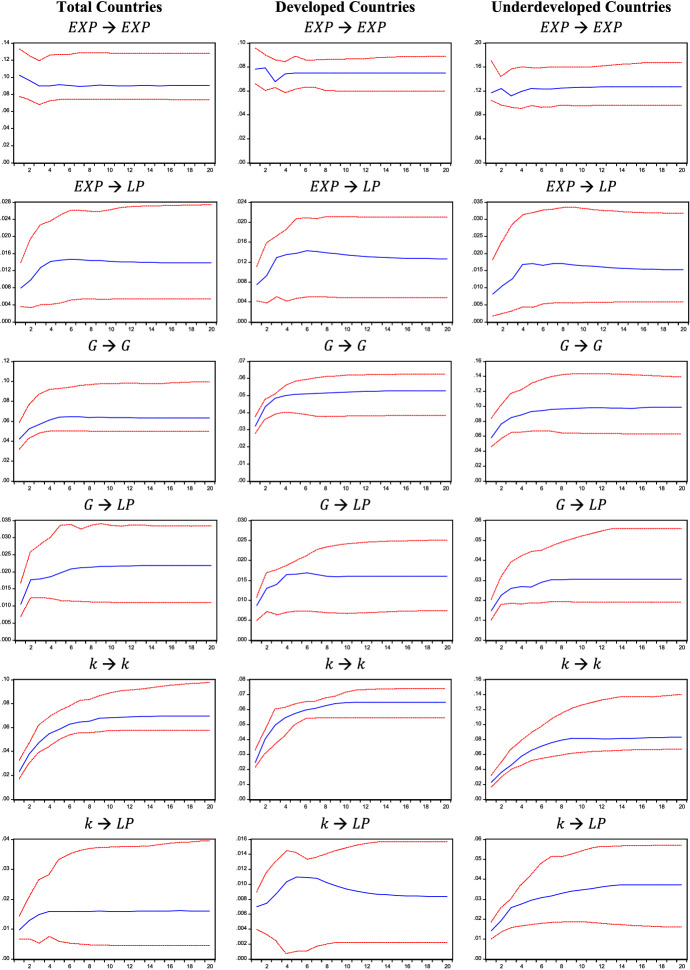
Impulse response functions (IRFs), Model 3. IRFs are reported with two standard error bounds (95% confidence interval)

## Data Availability

The data described in this article are openly available at https://www.rug.nl/ggdc/productivity/pwt/.

## Appendix A: Data Sources

Gross Domestic Product ($$Y$$): Output-side real GDP at current PPPs (in mil. 2017US$). Penn World Table, version 10.0. Variable CGDPO. Available at: https://www.rug.nl/ggdc/docs/pwt100.xlsx

Capital-labour ratio ($$k$$): Capital stock at current PPPs (in mil. 2017US$) (CN) divided by Average annual hours worked by persons engaged (AVH). Penn World Table, version 10.0. Variables: CN and AVH respectively in the original dataset. Available at: https://www.rug.nl/ggdc/docs/pwt100.xlsx

Labour Productivity ($$LP$$): Output-side real GDP at current PPPs (in mil. 2017US$) divided by average annual hours worked by persons engaged. Variables: CGDPO and AVH respectively in the original dataset. Available at: https://www.rug.nl/ggdc/docs/pwt100.xlsx

Government Consumption ($$G$$): Output-side real GDP at current PPPs (in mil. 2017US$) multiplied by share of government consumption at current PPPs. Variables: CGDPO and CSH_G respectively in the original dataset. Available at: https://www.rug.nl/ggdc/docs/pwt100.xlsx

Exports ($$EXP$$): Output-side real GDP at current PPPs (in mil. 2017US$) multiplied by share of merchandise exports at current PPPs. Variables: CGDPO and CSH_X respectively in the original dataset. Available at: https://www.rug.nl/ggdc/docs/pwt100.xlsx

Autonomous components ($Z$): Government Consumption ($G$) plus Exports ($EXP$).

## Appendix B

A robustness check is carried out on Model 3 by assuming a different identification strategy shown in Eq. (10), where government expenditure is considered more exogenous than export in the contemporaneous relationship. Estimated IRFs and cumulative effects are reported in Fig. 4 and Table 2, respectively. As in the main model specification, IRFs are highly persistent as they remain positive throughout the 20-year period. Furthermore, cumulative effects are similar to the findings obtained in Sect. 3, thus showing that findings are robust to different model specifications.

10 $$\mathbf{Model\;3{:}} \quad {{B}_{0i}y}_{it}=\left[\begin{array}{cccc}-& 0& 0& 0\\ -& -& 0& 0\\ -& -& -& 0\\ -& -& -& -\end{array}\right]\left[\begin{array}{c}{G}_{i,t}\\ {EXP}_{i,t}\\ {k}_{i,t}\\ {LP}_{i,t}\end{array}\right]$$

**Table 2:** Cumulative effects

	1 year	5 years	10 years	20 years	Average
All countries					
$G$	**0.249**	**0.306**	**0.339**	**0.346**	0.331
$EXP$	**0.076**	**0.158**	**0.157**	**0.153**	0.148
$k$	**0.423**	**0.270**	**0.232**	**0.230**	0.258
Developed					
$G$	**0.272**	**0.325**	**0.306**	**0.304**	0.307
$EXP$	**0.095**	**0.184**	**0.179**	**0.169**	0.170
$k$	**0.284**	**0.190**	**0.145**	**0.128**	0.158
Developing					
$G$	**0.254**	**0.288**	**0.312**	**0.309**	0.306
$EXP$	**0.069**	**0.137**	**0.130**	**0.120**	0.122
$k$	**0.627**	**0.452**	**0.424**	**0.446**	0.463

Models 3. Significant effects are in bold

## Footnotes

1 Another strand of literature explains the stagnation of productivity dynamics through the concept of ‘Structural Change’. According to this perspective, which starts with Baumol’s (1967) seminal work, economies would experience stagnating productivity due to an expansion of the service sector—compared to other economic sectors—which is associated with lower productivity dynamics and potential productivity gains compared to other sectors (e.g., the manufacturing sector) (Storm, 2017; Szirmai and Verspagen, 2015). However, such a perspective has been critically discussed by Deleidi et al. (2020b) who have proved that stagnating productivity is mainly ascribable to a slowdown in aggregate demand rather than shifts toward the service sector, and Fontanari and Palumbo (2022) who have also emphasized the role of stagnating wages.

2 The role of embodied technical progress is also analysed by several scholars from different schools of thought (see, among others, Barbieri et al., 2019; Dosi et al., 2021; Nomaler et al., 2021). For a multisectoral analysis, see Aroche Reyes (2021).

3 The supermultiplier model accounts for both the multiplier and accelerator effects. The latter is based on a flexible adjustment, namely on the idea that firms adjust investment to demand gradually.

4 The Supermultiplier model of growth was developed by Serrano (1995) and Bortis (1997) at the end of the nineties and in recent years it has gained momentum in the international debate among scholars with different theoretical backgrounds (see, among others, Allain, 2015; Deleidi and Mazzucato, 2019, 2021; Fazzari et al., 2020; Fiebiger and Lavoie, 2019; Lavoie, 2016; Palley, 2019).

5 It should be notices that, recently, de Oliveira and Lima (2022) further develop the Kaldor–Verdoorn law taking into account the effects on the environment. According to these authors, an increase in pollution concentration leading to environmental degradation might thus offset the productivity-enhancing effect of output growth.

6 Additional studies have also provided evidence in favour of the role of demand in stimulating innovation. See, among others, Bogliacino and Pianta (2013), Guarascio et al. (2016), and Crespi and Guarascio (2019).

7 For an in-depth review of the theories and causes of the Italian regional disparities in labour productivity growth, see Bartoloni et al., (2022a, 2022b) and Deleidi et al. (2021b), among others. Furthermore, for an analysis on the role of public and private investments to explain the Italian economic development, see Garegnani (2015).

8 The final data contains the following countries: Argentina (1950–2019), Australia (1950–2019), Austria (1950–2019), Bangladesh (1970–2019), Belgium (1950–2019), Brazil (1950–2019), Canada (1950–2019), Chile (1951–2019), China (1970–2019), China Hong Kong (1960–2019), Colombia (1950–2019), Costa Rica (1987–2019), Denmark (1950–2019), Dominican Republic (1990–2019), Finland (1950–2019), France (1950–2019), Germany (1950–2019), Greece (1951–2019), Hungary (1980–2019), Iceland (1964–2019), India (1970–2019), Indonesia (1970–2019), Ireland (1950–2019), Israel (1981–2019), Italy (1950–2019), Japan (1950–2019), Luxembourg (1970–2019), Malaysia (1970–2019), Mexico (1950–2019), Myanmar (1970–2019), Netherlands (1950–2019), New Zealand (1970–2019), Norway (1950–2019), Pakistan (1970–2019), Peru (1950–2019), Philippines (1970–2019), Portugal (1950–2019), Republic of Korean (1953–2019), Singapore (1960–2019), Slovakia (1990–2019), Spain (1950–2019), Sri Lanka (1970–2019), Sweden (1950–2019), Switzerland (1950–2019), Taiwan (1951–2019), Thailand (1970–2019), Turkey (1970–2019), United Kingdom (1950–2019), United States (1950–2019), Uruguay (1990–2019), Venezuela (1950–2006) and Viet Nam (1970–2019).

9 In the current paper, we do not want to focus on the cyclical fluctuations of labour productivity as instead postulated by Okun’s law. However, for an in-depth analysis of this topic, see, Okun (1962), Jeon and Vernengo (2007) and Fontanari et al. (2020), among others.

10 For the sake of simplicity, IRFs corresponding with composite shocks are reported in Sect. 5, while those associated with common and idiosyncratic shocks are available upon request.

11 All variables are taken at levels to preserve any cointegrating or long-run relationship that may exist among the considered variables (Auerbach and Gorodnichenko, 2012, ft. 6; Kilian and Lütkepohl, 2017).

12 Such identification allows us to solve a debatable issue raised in the empirical literature grounded on the Kaldorian framework, according to which capital accumulation is found not significant in affecting labour productivity when using a single equation modelling since output dynamics endogenously determine investment and capital following the accelerator principle.

13 According to Kaldor (1975b), the tendency displayed by industrial sectors in advanced economies to engage in cumulative causation processes of growth triggered by exports underlies the world’s growing polarisation between rich and poor areas.

## References

[CR1] Allain O (2015). Tackling the instability of growth: A Kaleckian–Harrodian model with an autonomous expenditure component. Cambridge Journal of Economics.

[CR2] Antenucci F, Deleidi M, Paternesi Meloni W (2020). Kaldor 3.0: An empirical investigation of the Verdoorn-augmented technical progress function. Review of Political Economy.

[CR3] Apergis N, Zikos S (2003). The law of Verdoorn: Evidence from Greek disaggregated manufacturing time series data. The Economic and Social Review.

[CR4] Aroche Reyes F (2021). La ley de Kaldor–Verdoorn desde una perspectiva multisectorial. Cuadernos De Economía.

[CR5] Arrow KJ (1971). The economic implications of learning by doing. The Review of Economic Studies.

[CR6] Auerbach AJ, Gorodnichenko Y (2012). Measuring the output responses to fiscal policy. American Economic Journal: Economic Policy.

[CR7] Barbieri L, Piva M, Vivarelli M (2019). R&D, embodied technological change, and employment: Evidence from Italian microdata. Industrial and Corporate Change.

[CR8] Barbieri Góes MC, Deleidi M (2022). Output determination and autonomous demand multipliers: An empirical investigation for the US economy. Economic Modelling.

[CR9] Bartoloni, E., Baussola, M., Marino, A., & Romaniello, D. (2022a). The productivity puzzle: Firms, workers, and industry characteristics. *Vita e Pensiero*, Quaderno n. 157/aprile 2022a.

[CR10] Bartoloni, E., Baussola, M., Marino, A., & Romaniello, D. (2022b). Urban non-urban agglomeration divide: Is there a gap in productivity and wages? *Italian Economic Journal*, 1–39.

[CR1000] Baumol WJ (1967). Macroeconomics of unbalanced growth: the anatomy of urban crisis. The American economic review.

[CR11] Bianchi C (2002). A reappraisal of Verdoorn’s law for the Italian economy, 1951–1997. Productivity growth and economic performance.

[CR12] Blanchard O, Perotti R (2002). An empirical characterization of the dynamic effects of changes in government spending and taxes on output. The Quarterly Journal of Economics.

[CR13] Bogliacino F, Pianta M (2013). Profits, R&D, and innovation—A model and a test. Industrial and Corporate Change.

[CR14] Borsato A, Lorentz A (2022). The Kaldor–Verdoorn law’s at the age of robots and AI.

[CR15] Bortis H (1997). Institutions, behaviour and economic theory: A contribution to classical-Keynesian political economy.

[CR16] Carnevali E, Godin A, Lucarelli S, Veronese Passarella M (2020). Productivity growth, Smith effects and Ricardo effects in Euro area's manufacturing industries. Metroeconomica.

[CR17] Castiglione C (2011). Verdoorn–Kaldor's law: An empirical analysis with time series data in the United States. Advances in Management and Applied Economics.

[CR18] Cesaratto S, Serrano F, Stirati A (2003). Technical change, effective demand and employment. Review of Political Economy.

[CR19] Clavijo-Cortes, P. (2021). Output hysteresis in the US: New evidence from a time-varying Verdoorn's law. *Journal of Economic Studies*.

[CR20] Crespi F, Guarascio D (2019). The demand-pull effect of public procurement on innovation and industrial renewal. Industrial and Corporate Change.

[CR21] de Oliveira G, Lima GT (2022). Economic growth as a double-edged sword: The pollution-adjusted Kaldor–Verdoorn effect. Ecological Economics.

[CR22] Deleidi, M., Iafrate, F., & Levrero, E. S. (2021a). Government investment fiscal multipliers: Evidence from Euro-area countries. *Macroeconomic Dynamics*, 1–19.

[CR23] Deleidi M, Iafrate F, Levrero ES (2020). Public investment fiscal multipliers: An empirical assessment for European countries. Structural Change and Economic Dynamics.

[CR24] Deleidi M, Mazzucato M (2019). Putting austerity to bed: Technical progress, aggregate demand and the supermultiplier. Review of Political Economy.

[CR25] Deleidi M, Mazzucato M (2021). Directed innovation policies and the supermultiplier: An empirical assessment of mission-oriented policies in the US economy. Research Policy.

[CR27] Deleidi, M., Meloni, W. P., Salvati, L., & Tosi, F. (2021b). Output, investment and productivity: The Italian North–South regional divide from a Kaldor–Verdoorn approach. *Regional Studies*, 1–12.

[CR26] Deleidi M, Meloni WP, Stirati A (2020). Tertiarization, productivity and aggregate demand: Evidence-based policies for European countries. Journal of Evolutionary Economics.

[CR28] Deleidi, M., Romaniello, D., & Tosi, F. (2021c). Quantifying fiscal multipliers in Italy: A panel SVAR analysis using regional data. *Papers in Regional Science*.

[CR29] Dosi G, Piva M, Virgillito ME, Vivarelli M (2021). Embodied and disembodied technological change: The sectoral patterns of job-creation and job-destruction. Research Policy.

[CR30] Dosi G, Yu X (2019). Technological catching-up, sales dynamics, and employment growth: Evidence from China’s manufacturing. Industrial and Corporate Change.

[CR31] Fazzari SM, Ferri P, Variato AM (2020). Demand-led growth and accommodating supply. Cambridge Journal of Economics.

[CR32] Feenstra RC, Inklaar R, Timmer MP (2015). The next generation of the Penn World Table. American Economic Review.

[CR33] Fiebiger B, Lavoie M (2019). Trend and business cycles with external markets: Non-capacity generating semi-autonomous expenditures and effective demand. Metroeconomica.

[CR34] Foda, K. (2017). What’s happening to productivity growth? Key macro trends and patterns.

[CR35] Fontanari C, Palumbo A (2022). Permanent scars: The effects of wages on productivity. Metroeconomica.

[CR36] Fontanari C, Palumbo A, Salvatori C (2020). Potential output in theory and practice: A revision and update of Okun's original method. Structural Change and Economic Dynamics.

[CR37] Forges Davanzati G, Patalano R, Traficante G (2019). The Italian economic stagnation in a Kaldorian theoretical perspective. Economia Politica.

[CR38] Freitas F, Serrano F (2015). Growth rate and level effects, the stability of the adjustment of capacity to demand and the Sraffian supermultiplier. Review of Political Economy.

[CR39] Gabrisch H (2021). The long-run properties of the Kaldor–Verdoorn law: A bounds test approach to a panel of Central and East European (CEE) countries. Empirica.

[CR40] Garegnani P (2015). The problem of effective demand in Italian economic development: On the factors that determine the volume of investment. Review of Political Economy.

[CR41] Guarascio D, Pianta M, Bogliacino F (2016). Export, R&D and new products. A model and a test on European industries. Journal of Evolutionary Economics.

[CR42] Iasco Pereira HC, Romero JP, Medeiros V (2021). Kaldor–Verdoorn’s law and institutions: Evidence from Brazilian municipalities. Cambridge Journal of Economics.

[CR43] International Monetary Fund (IMF) (2020). Fiscal monitor: Policies for the recovery.

[CR44] Jeon Y, Vernengo M (2007). Puzzles paradoxes and regularities: Cyclical and structural productivity in the US (1950–2005).

[CR45] Jorgenson DW, Ho MS, Stiroh KJ (2008). A retrospective look at the US productivity growth resurgence. Journal of Economic Perspectives.

[CR46] Kaldor N (1957). A model of economic growth. Economic Journal.

[CR47] Kaldor, N. (1966) Causes of the slow rate of economic growth of the United Kingdom: An inaugural lecture. Cambridge University Press. Reprinted in N. Kaldor (1978), Further essays on economic theory, London: Duckworth.

[CR48] Kaldor N (1968). Productivity and growth in manufacturing industry: A reply. Economica.

[CR49] Kaldor N (1970). The case for regional policies. Scottish Journal of Political Economy.

[CR50] Kaldor N (1972). The irrelevance of equilibrium economics. The Economic Journal.

[CR51] Kaldor, N (1975a) Economic growth and Verdoorn`s law. A comment on Mr. Routhorn’s Article. *Economic Journal*, *85*.

[CR52] Kaldor N (1975). What is wrong with economic theory. Quarterly Journal of Economics.

[CR53] Kaldor N (1977). Capitalism and industrial development: Some lessons from Britain's experience. Cambridge Journal of Economics.

[CR54] Kaldor N, Mirrlees JA (1962). A new model of economic growth. The Review of Economic Studies.

[CR55] Kilian L, Lütkepohl H (2017). Structural vector autoregressive analysis.

[CR56] Knell M (2004). Structure change and the Kaldor–Verdoorn law in the 1990s. Revue D'économie Industrielle.

[CR57] Kumar S, Russell RR (2002). Technological change, technological catch-up, and capital deepening: Relative contributions to growth and convergence. American Economic Review.

[CR58] Lavoie M (2015). Post-Keynesian economics: New foundations.

[CR59] Lavoie M (2016). Convergence towards the normal rate of capacity utilization in neo-Kaleckian models: The role of non-capacity creating autonomous expenditures. Metroeconomica.

[CR60] Magacho GR, McCombie JS (2017). Verdoorn’s law and productivity dynamics: An empirical investigation into the demand and supply approaches. Journal of Post Keynesian Economics.

[CR61] Magacho GR, McCombie JS (2018). A sectoral explanation of per capita income convergence and divergence: Estimating Verdoorn’s law for countries at different stages of development. Cambridge Journal of Economics.

[CR62] McCombie JSL (1983). Kaldor’s laws in retrospect. Journal of Post Keynesian Economics.

[CR63] McCombie J, Pugno M, Soro B (2002). Productivity growth and economic performance: Essays on Verdoorn's law.

[CR64] Michl TR (1985). International comparisons of productivity growth: Verdoorn’s law revisited. Journal of Post Keynesian Economics.

[CR65] Millemaci, E., & Ofria, F. (2014). Kaldor–Verdoorn's law and increasing returns to scale: A comparison across developed countries. *Journal of Economic Studies*.

[CR66] Nah WJ, Lavoie M (2019). Convergence in a neo-Kaleckian model with endogenous technical progress and autonomous demand growth. Review of Keynesian Economics.

[CR67] Nassif A, Feijó C, Araujo E (2022). Econometric estimation of labor productivity in the Brazilian Manufacturing Sector in the 2000s: A Kaldorian approach. Revista Brasileira De Inovação.

[CR68] Nomaler Ö, Spinola D, Verspagen B (2021). R&D-based economic growth in a supermultiplier model. Structural Change and Economic Dynamics.

[CR69] Ofria F (2009). L'approccio Kaldor–Verdoorn: una verifica empirica per il Centro-Nord e il Mezzogiorno d'Italia (anni 1951–2006). Rivista Di Politica Economica.

[CR70] Okun AM (1962). The predictive value of surveys of business intentions. The American Economic Review.

[CR71] Palley T (2019). The economics of the super-multiplier: A comprehensive treatment with labor markets. Metroeconomica.

[CR72] Palumbo A (2009). Adjusting theory to reality: The role of aggregate demand in Kaldor's late contributions on economic growth. Review of Political Economy.

[CR73] Pedroni P (2013). Structural panel VARs. Econometrics.

[CR74] Serrano F (1995). Long period effective demand and the Sraffan supermultiplier. Contributions to Political Economy.

[CR76] Setterfield M, Cornwall J, Setterfield M (2002). A neo-Kaldorian perspective on the rise and decline of the Golden Age. The economics of demand-led growth.

[CR77] Sichel, D. E., & Oliner, S. D. (2002). Information technology and productivity: Where are we now and where are we going?. *Available at SSRN 318692*.

[CR78] Smith, A. (1776). *An inquiry into the nature and causes of the wealth of nations*. Edited by A. Skinner, Harmondsworth: Penguin Books, 1973. Cap, I, II, III.

[CR79] Spilimbergo, A., Symansky, S., & Schindler, M. (2009). Fiscal multipliers. *IMF Staff Position Notes*, *2009*(011).

[CR80] Storm S (2017). The new normal: Demand, secular stagnation, and the vanishing middle class. International Journal of Political Economy.

[CR81] Sylos-Labini P (1984). Le forze dello sviluppo e del declino.

[CR82] Szirmai A, Verspagen B (2015). Manufacturing and economic growth in developing countries, 1950–2005. Structural Change and Economic Dynamics.

[CR83] Thirlwall AP (1979). The balance of payments constraint as an explanation of international growth rate differences. BNL Quarterly Review.

[CR85] Thirlwall AP (1983). Foreign trade elasticities in centre-periphery models of growth and development. BNL Quarterly Review.

[CR86] Tridico P, Pariboni R (2018). Inequality, financialization, and economic decline. Journal of Post Keynesian Economics.

[CR87] United Nations (UN). (2020). World economic situation and prospects, Statistical Annex.

[CR88] Van Ark B, O'Mahoney M, Timmer MP (2008). The productivity gap between Europe and the United States: Trends and causes. Journal of Economic Perspectives.

[CR89] Verdoorn JP (1949). On the factors determining the growth of labor productivity. Italian Economic Papers.

